# Editorial: Recent advances in the research and development of kinase-inhibitory anticancer molecules

**DOI:** 10.3389/fchem.2023.1328424

**Published:** 2023-11-09

**Authors:** Ahmed A. Al-Karmalawy, Hussein I. El-Subbagh, Liliya Logoyda, Roman B. Lesyk, Mohammed I. El-Gamal

**Affiliations:** ^1^ Department of Pharmaceutical Chemistry, Faculty of Pharmacy, Horus University-Egypt, New Damietta, Egypt; ^2^ Pharmaceutical Chemistry Department, Faculty of Pharmacy, Ahram Canadian University, Giza, Egypt; ^3^ Department of Medicinal Chemistry, Faculty of Pharmacy, Mansoura University, Mansoura, Egypt; ^4^ Pharmacy Center of Scientific Excellence, Faculty of Pharmacy, Mansoura University, Mansoura, Egypt; ^5^ Department of Pharmaceutical Chemistry, I. Horbachevsky Ternopil National Medical University, Ternopil, Ukraine; ^6^ Department of Pharmaceutical, Organic, and Bioorganic Chemistry, Danylo Halytsky Lviv National University, Lviv, Ukraine; ^7^ Department of Medicinal Chemistry, College of Pharmacy, University of Sharjah, Sharjah, United Arab Emirates; ^8^ Research Institute for Medical and Health Sciences, University of Sharjah, Sharjah, United Arab Emirates

**Keywords:** protein kinases, kinase inhibitors, rational design, multitargeted kinase inhibitors, anticancer

Protein kinases (PKs) represent one of the most important targets in the discovery of new drug candidates in oncology based on their crucial roles in the processes of cellular growth and proliferation (Fabbro et al., 2002). Kinase inhibitors are classified into 1) type I kinase inhibitors (interacting directly with the ATP binding site), 2) type II kinase inhibitors (interacting with the ATP binding site besides extra hydrophobic interactions to adjacent hydrophobic pocket), 3) type III kinase inhibitors or allosteric inhibitors (interacting with a hydrophobic pocket distant from the ATP binding site), and 4) others (such as covalent and protein substrate competitive inhibitors) (Chahrour et al., 2012).

However, most of the currently available kinase inhibitors were recorded to be less effective over time, especially in the treatment of complex and mutagenic tumors due to their design to act on a single target. On the contrary, multitargeted kinase inhibitors were observed to be more effective due to their ability to inhibit multiple pathways in cancer cell proliferation which results in decreasing the possibility of drug resistance development (Guo and Ma, 2021; El-Naggar et al., 2022).

Herein, four research articles were published on this Research Topic received from China, Sudan, Saudi Arabia, and Germany. https://www.frontiersin.org/research-topics/33242/recent-advances-in-the-research-and-development-of-kinase-inhibitory-anticancer-molecules.

Not only synthetic medicinal chemistry contributes to the discovery and development of kinase inhibitors, many natural compounds also have been reported as kinase inhibitors. In the original research article of Tian et al., inhibitors isolated from *Zanthoxylum simulans* were reported as inhibitors of multiple janus kinases (JAKs). The authors developed JAKs based affinity ultrafiltration method coupled with LC/Q-TOF-MS to enable them to study the affinity of *Z. simulans* quaternary alkaloids against JAKs. They discovered that Berberine and Chelerythrine exhibited superior selectivity against JAK1, JAK2, and JAK3 over Tyk2. Chelerythrine demonstrated promising antiproliferative activity against AGS gastric cancer cells. Both Berberine and Chelerythrine showed obvious inhibition against LO2 human hepatocyte cells. Chelerythrine-mediated inhibition and apoptosis against AGS cells happened via the Estrogen Pathway. This is in addition to direct inhibition of JAK1, JAK2, and JAK3 kinases. The authors studied the putative binding interactions of the quaternary alkaloids to reveal the reasons for the selectivity towards the three JAK isozymes.

The other article authored by Mohamed et al. reported another contribution of natural products to kinase inhibitors discovery. *In silico* studies including molecular docking, molecular dynamic simulation, and pharmacophore modeling led to discovery of dual MNK/PIM as potential antiproliferative candidates for treatment of acute myeloid leukemia. The authors started their study with pharmacophore modeling of ligands bound to MNK-2 and PIM-2 crystal structures, and the obtained pharmacophoric features were screened against 270,540 natural compounds from ZINC database. The matched natural compounds were docked into the binding sites of MNK-2 and PIM-2 kinases.

The original research article by Fu et al. reported the design and synthesis of a series of flavone-based cyclin-dependent kinase 1 (CDK1) inhibitors. The most potent CDK1 inhibitor among this series exhibited higher inhibitory activity against RAW 264.7 than MCF-7 cells. This hit compound can be useful for further optimization towards antiproliferative and anti-inflammatory candidates development. The authors studied the putative binding interactions of the most active inhibitor with CDK1 crystal structure.

The fourth published article entitled Heine et al. (*Spoilt for choice: different immunosuppressive potential of anaplastic lymphoma kinase inhibitors for non-small cell lung cancer*), where the authors investigated the effect of alectinib and crizotinib on human monocyte-derived DCs (moDC) as the most powerful antigen-presenting cells. Crizotinib-treated DCs were observed to reduce the activation markers such as CD83, chemokine-guided migration, antigen uptake, and pro-inflammatory cytokines, especially Interleukin-12. These effects were superior to those of alectinib and therefore crizotinib could dampen the anti-tumor immunity more effectively. Briefly, crizotinib alone was confirmed to have immunosuppressive effects on DCs phenotype and reduced DC function, accordingly potentially impairing anti-tumor immunity.
